# How Does Explanatory Virtue Determine Probability Estimation?—Empirical Discussion on Effect of Instruction

**DOI:** 10.3389/fpsyg.2020.575746

**Published:** 2020-12-09

**Authors:** Asaya Shimojo, Kazuhisa Miwa, Hitoshi Terai

**Affiliations:** ^1^Department of Cognitive and Psychological Sciences, Graduate School of Informatics, Nagoya University, Nagoya, Japan; ^2^Department of Information and Computer Science, Faculty of Humanity-Oriented Science and Engineering, Kindai University, Higashi-osaka, Japan

**Keywords:** causal explanation, diagnostic reasoning, subjective probability, explanatory virtue, inference to the best explanation

## Abstract

It is important to reveal how humans evaluate an explanation of the recent development of explainable artificial intelligence. So, what makes people feel that one explanation is more likely than another? In the present study, we examine how explanatory virtues affect the process of estimating subjective posterior probability. Through systematically manipulating two virtues, Simplicity—the number of causes used to explain effects—and Scope—the number of effects predicted by causes—in three different conditions, we clarified two points in Experiment 1: (i) that Scope's effect is greater than Simplicity's; and (ii) that these virtues affect the outcome independently. In Experiment 2, we found that instruction about the explanatory structure increased the impact of both virtues' effects but especially that of Simplicity. These results suggest that Scope predominantly affects the estimation of subjective posterior probability, but that, if perspective on the explanatory structure is provided, Simplicity can also affect probability estimation.

## Introduction

Humans are explanatory creatures (Norman, 1988). Whenever we seek to determine whether, for example, a patient has a disease, or whether a defendant is guilty of a crime, humans desire an explanation that will reveal the cause of the observed events (Lipton, 2002).

### Psychological Research About Evaluating Explanations

What aspects of these causal explanations make us feel that an explanation is more likely than the others?

The Bayesian approach in cognitive science assumes that humans update their beliefs based on a rule called Bayes' Rule (Phillips and Edwards, 1966), which states that:

(1) $$\begin{array}{l} P\left(C|E\right)=\frac{P\left(E|C\right)P\left(C\right)}{P\left(E\right)} \end{array}$$

According to this rule, when humans observe an event *E*, they use the probability of *E* under another independent event *C* (henceforth, “likelihood”) *P(E|C)* to update their preconceived probability of *C*'s occurrence (henceforth, “prior probability”) *P(C)* and to infer the probability that *C* is the cause of *E* (henceforth, “posterior probability”) *P(C|E)*. In psychological research, posterior probability is considered a subjective probability; in this study, it is called “subjective posterior probability” because it expresses the degree of a cognitive subject's confidence in their conclusions about the causes that have led to an observed event.

For instance, suppose that a reagent tests to determine whether or not a subject uses a certain drug. Consider the probability that the positive result is caused by the drug if a subject takes this test and its result reveals positive. In this case, the probability that the subject uses the drug before the test was performed is, *P(C)*, i.e., the probability that the drug is used in that society in the first place. This knowledge, *P(C)*, we can know prior to the test is called the prior probability. If the test is then performed and the reagent tests positive, the knowledge we have is updated. Specifically, *P(C|E)* is calculated by Equation (1) using *P(E)*, the probability that the reagent tests positive, and *P(E|C)*, the probability that the reagent tests positive when the drug is indeed used. This is called the posterior probability, meaning that the new information by the test allows us to calculate a more accurate probability than the prior probability. In summary, the Bayesian approach is to update the probability that a cause induced an event after taking into consideration new information of the event. We use the approach to reveal the probability that certain causes actually induced observed events based on observed results. This approach has been widely used in various domains, ranging from human everyday reasoning through determining false positives in clinical trials and the filtering junk e-mail.

In practice, however, humans tend to estimate subjective posterior probability according to schemes that deviate from Bayes' rule (Tversky and Kahneman, 1974; Koehler, 1993; Barbey and Sloman, 2007). In the context of causal explanations, Lipton (2002) argues that explanatory goodness leads to subjective posterior probability; more recently, other studies have reported results that support this proposal (Douven and Schupbach, 2015; Johnson et al., 2016c; Douven and Mirabile, 2018). Specifically, the notion of “explanatory virtue,” which shapes our perceptions of the goodness of an explanation, has been examined experimentally to see how it affects subjective posterior probability and the estimation of the probability information (Prior probability, likelihood) needed to derive it. For instance, William of Occam's assertion (Occam's razor; Jefferys and Berger, 1992) that we should not assume more causes than necessary to explain an event is consistent with the explanatory virtue known as Simplicity; Lombrozo (2007) has experimentally examined the relationship between Simplicity and Prior probability, and found that humans tend to prefer explanations with low Prior probability but fewer causes to assume.

Other candidates for explanatory virtue are also known such as Scope, Consistency, and Fruitfulness (Lombrozo, 2007). Specifically, Scope is a virtue regarding the number of events being explained. Consistency is a virtue regarding whether there is a contradiction between the participants' prior knowledge and the causal relationship explained (Dawes, 2012), and Fruitfulness is a virtue regarding how many relationships, which participants previously unnoticed between the cause and the event being explained, can be given to them (Kuhn, 1977). Consistency and Fruitfulness are virtues mainly related to the contents of an explanation, whereas Scope, like Simplicity, is a virtue about causal structures of explanations. As shown below, among these explanatory virtues, there has been a lot of research conducted on Scope specifically. Johnson et al. (2014b) found that explanations that correctly predict more events (i.e., those with wider Scope) make us feel more certain than explanations that predict fewer events. On the other hand, it has been reported that explanations that predict more events but that include causes that predict unobserved events are perceived as having lower subjective posterior probability than explanations that do not include causes that predict unobserved events (Khemlani et al., 2011; Johnson et al., 2014b, 2016c).

All of these studies, however, have examined Simplicity and Scope separately, as distinct factors contributing to explanatory virtue. Therefore, they have not attended to the magnitude of the effect of each factor on the estimation of subjective posterior probability or to the existence of an interaction between the two factors. In this study, we systematically manipulated both Simplicity and Scope to examine the degree of their effects and their interaction. In addition, assuming that humans calculate probability based in part on Bayes' Rule, one might expect that explanations with higher Prior probability would be perceived as having higher subjective posterior probability. Therefore, in this study, we also manipulated the Prior probability of an explanation as an independent variable in order to investigate its effect on the estimation of subjective posterior probability.

## Experiment 1

Two experiments in present study involving human participants was reviewed and approved by the Ethics Committee of Department of Cognitive and Psychological Sciences, Nagoya University. The approval number is NUPSY-190530-B-01.

This experiment consisted of three experimental situations, 1A, 1B, and 1C. In these experimental situations, we examined the extent to which Simplicity, Scope, and Prior probability affect the estimation of subjective posterior probability, focusing especially on the presence or absence of interaction between Simplicity and Scope.

### Definition of Explanatory Virtues

Explanatory virtue refers to features about a content of explanation (Lombrozo, 2016). Hence, it corresponds to the causal structure of explanation, such as the number of causes used to explain effects and the number of effects predicted by causes. The purpose of this study is to examine the influence of explanatory virtues, especially the ones referring to causal structure, on the estimation of the subjective posterior probability of explanations. Consistency and Fruitfulness are considered to be explanatory virtues that depend on the participants' prior knowledge because they are related to the contents of the explanation. The purpose of this study is to focus on the discussion about the explanatory virtues, especially the ones related to the causal structure rather than the contents of the explanations. Therefore, we deal with Simplicity and Scope as explanatory virtues relating to causal structures of explanation in this study.

In this paper, we define Simplicity and Scope as follows. First, Simplicity is quantified as the number of causes invoked in an explanation (Lombrozo, 2007). Thus we define an explanation with one cause (i.e., fewer causes) as “Simple” and an explanation with two causes (i.e., more causes) as “Complex.” Second, Scope is quantified as the number of effects invoked by cause(s) in an explanation (Johnson et al., 2014b). We further divide Scope into two parts: Manifest and Latent. Manifest scope indicates only observed effects (Read and Marcus-Newhall, 1993), whereas Latent scope also includes unobserved effects (Khemlani et al., 2011). Regarding Scope factor, we define an explanation with one effect predicted by causes (i.e., an explanation that can explain fewer effects) as “Narrow” and an explanation with two effects (i.e., an explanation that can explain more effects) as “Wide.” We also define an explanation without any unobserved effects as “Manifest” and an explanation with at least one unobserved effect as “Latent.” We compared explanations of these types in two ways in this experiment: comparison between explanations with Manifest scope only (as in Experiment 1A) and comparison between an explanation with Latent scope and one with Manifest scope (as in Experiments 1B & 1C).

### Method

#### Participants

In this experiment, 221 undergraduate students at Nagoya University participated. They did not major in psychology or medical science and were recruited during lectures. Moreover, 89 students in Experiment 1A (48 men and 41 women, *M*_*age*_ = 19.72 years, *SD* = 0.90), 66 students in Experiment 1B (49 men and 17 women, *M*_*age*_ = 19.88 years, *SD* = 0.88), and 66 students in Experiment 1C (50 men and 16 women, *M*_*age*_ = 19.59 years, *SD* = 0.88) participated in exchange for course credit. Participants were informed that even if they were in the middle of the experiment, they could cancel their participation if they if they experience anxiety during this experiment.

The sample size was determined using G^*^Power3.1. Specifically, it was obtained by setting the significance level *p* at 5%, the effect size *f* at 0.4, and the power of a test *Power(1–*β *error probability)* at 0.95. The results showed that the number of samples required by G^*^Power was met in all experimental situations and that the test power was above 0.8 (Exp. 1A: *Power(1–*β *error probability)* = 0.962, Exp. 1B: *Power(1–*β *error probability)* = 0.893, and Exp. 1C: *Power(1–*β *error probability)* = 0.910).

#### Design

In each experimental situation, participants were provided with four explanations with different causal structures in which Simplicity, Scope, and Prior probability were manipulated to assess the effect of each variable on the estimation of subjective posterior probability (see Table 1). More specific aims and methods for each experiment are as follows.

- Exp. 1A: We manipulated Scope along a spectrum from Wide to Narrow and Simplicity along a spectrum from Simple to Complex to examine the effect of each explanatory virtue on the estimation of subjective posterior probability. Table 1-Exp. 1A shows the all structures used in Exp. 1A. The upper end of the table represents structures with Wide, and the lower end represents structures with Narrow. Meanwhile the left side of the table represents structures with Simple, and the right side represents structures with Complex. Normatively, humans assign higher subjective posterior probability to explanations with Wide Scope than to explanations with Narrow Scope, and to explanations with Simple than to explanations with Complex.
- Exp. 1B: We manipulated Scope along a spectrum from Latent to Manifest and Simplicity along a spectrum from Simple to Complex to examine the effect of each explanatory virtue on the estimation of subjective posterior probability. Unlike Exp. 1C, in this condition the number of events predicted by each cause was constant (i.e., E1 and E_2_ are predicted). Table 1-Exp. 1B shows the all structures used in Exp. 1B. The upper end of the table represents structures with Manifest, and the lower end represents structures with Latent. Meanwhile the left side of the table represents structures with Simple, and the right side represents structures with Complex. Normatively, humans assign higher subjective posterior probability to explanations with Manifest Scope than to explanations with Latent Scope.
- Exp. 1C: We manipulated Scope along a spectrum from Latent to Manifest and Simplicity along a spectrum from Simple to Complex to examine the effect of both explanatory virtues on the estimation of subjective posterior probability. Unlike Exp. 1B, in this condition the number of events actually observed was constant (i.e., E1 is observed). Table 1 shows the all structures used in Exp. 1C. The upper end of the table represents structures with Manifest, and the lower end represents structures with Latent. Meanwhile the left side of the table represents structures with Simple, and the right side represents structures with Complex. Normatively, humans assign higher subjective posterior probability to explanations with Manifest than to explanations with Latent.

**Table 1:** Explanatory structures used in Experiment 1.

**Exp. 1A**		
**Scope**	**Simplicity**	
	**Simple**	**Complex**
**Wide**	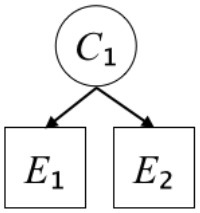	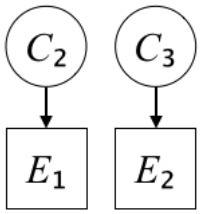
**Narrow**	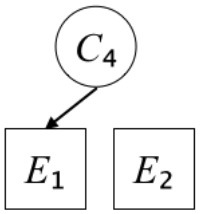	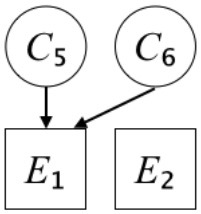
**Exp. 1B**		
**Scope**	**Simplicity**	
	**Simple**	**Complex**
**Manifest**	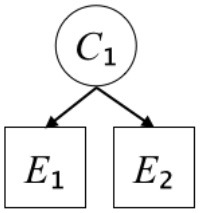	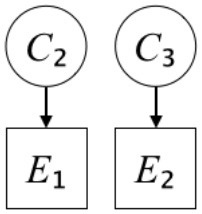
**Latent**	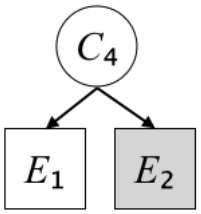	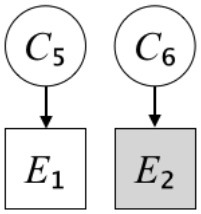
**Exp. 1C**		
**Scope**	**Simplicity**	
	**Simple**	**Complex**
**Manifest**	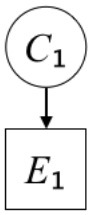	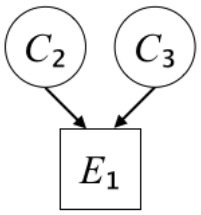
**Latent**	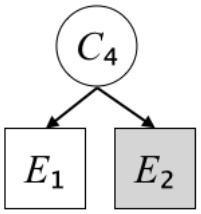	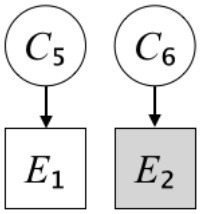

The independent variable in Experiment 1A was set to 2 (Simplicity: Simple, Complex; within-subject) × 2 (Scope: Wide, Narrow; within-subject) × 3 (Prior probability: 10, 20, 30%; between-subject). The independent variables in Experiments 1B and 1C were set to 2 (Simplicity: Simple, Complex; within-subject) × 2 (Scope: Latent, Manifest; within-subject) × 3 (Prior probability: 10, 20, 30%; between-subject). In all experimental situations, subjective posterior probability was used as the dependent variable.

#### Materials and Procedure

In this experiment, each participant was presented with a series of medical diagnosis scenarios as the task. In these scenarios, as in Johnson et al. (2014b), we used different symptoms as effects in each explanation in order to focus only on the structure of the explanation. Also, participants were randomly assigned one of the three levels of Prior probability (10, 20, 30%).

The experiment was carried out using a six-page questionnaire. On page 1, participants were informed that their role was to diagnose patients as a physician and assess the probability that each of them had the suggested disease. They were also told that it was impossible to calculate a mathematically accurate probability from the given information. On pages 2–5, four scenarios consisting of observed fictitious symptoms (Effects) were presented along with the names of fictitious diseases suggested to have caused the symptoms (Causes). The elements of the explanatory structure (i.e., Simplicity, Scope, Prior probability) were manipulated throughout the scenario. The following is an example of a scenario with a Simple and Wide explanation (30% Prior probability).

“*Watson visited you to seek medical advice. Today, a disease called “Toketsu-Byo,” is prevalent. It is confirmed that the incidence rate of the disease is approximately 30%. If an individual has the disease, symptoms E and F are observed. Through your examination of Watson, you found both symptoms E and F.”*

The following is an example of a scenario with a Simple and Latent explanation (30% Prior probability).

“*James visited you to seek medical advice. Today, a disease, called “Seiketsu-Byo,” is prevalent. It is confirmed that the incidence rate of the disease is approximately 30%. If an individual has the disease, symptoms A and B are observed. Through your examination of James, you found symptom A. However, you could not know whether James had symptom B or not because of a lack of examination equipment.”*

The order of the four explanations was randomized. In addition, as in Lagnado (1994), when two causes are assumed, the joint probability of the two causes was presented as the Prior probability.

After reading the descriptions on each page, the participants assessed the probability that the patient had the disease and recorded their responses on the questionnaire. Five minutes were assigned for each page. Specifically, participants were asked “How likely do you think it is that the patient has contracted the disease?” and reported their confidence regarding each of the four explanations (subjective posterior probability) on a scale of 0–100%. While working on each page, participants were not allowed to turn to any other page.

Finally, the participants reread each of the four explanations on pages 2–5 and re-evaluated the subjective posterior probabilities they had provided on each page. During this reevaluation, they were allowed to revise the subjective posterior probability of each explanation. The final assessments were recorded on page 6.

### Results

**Figure 1:**
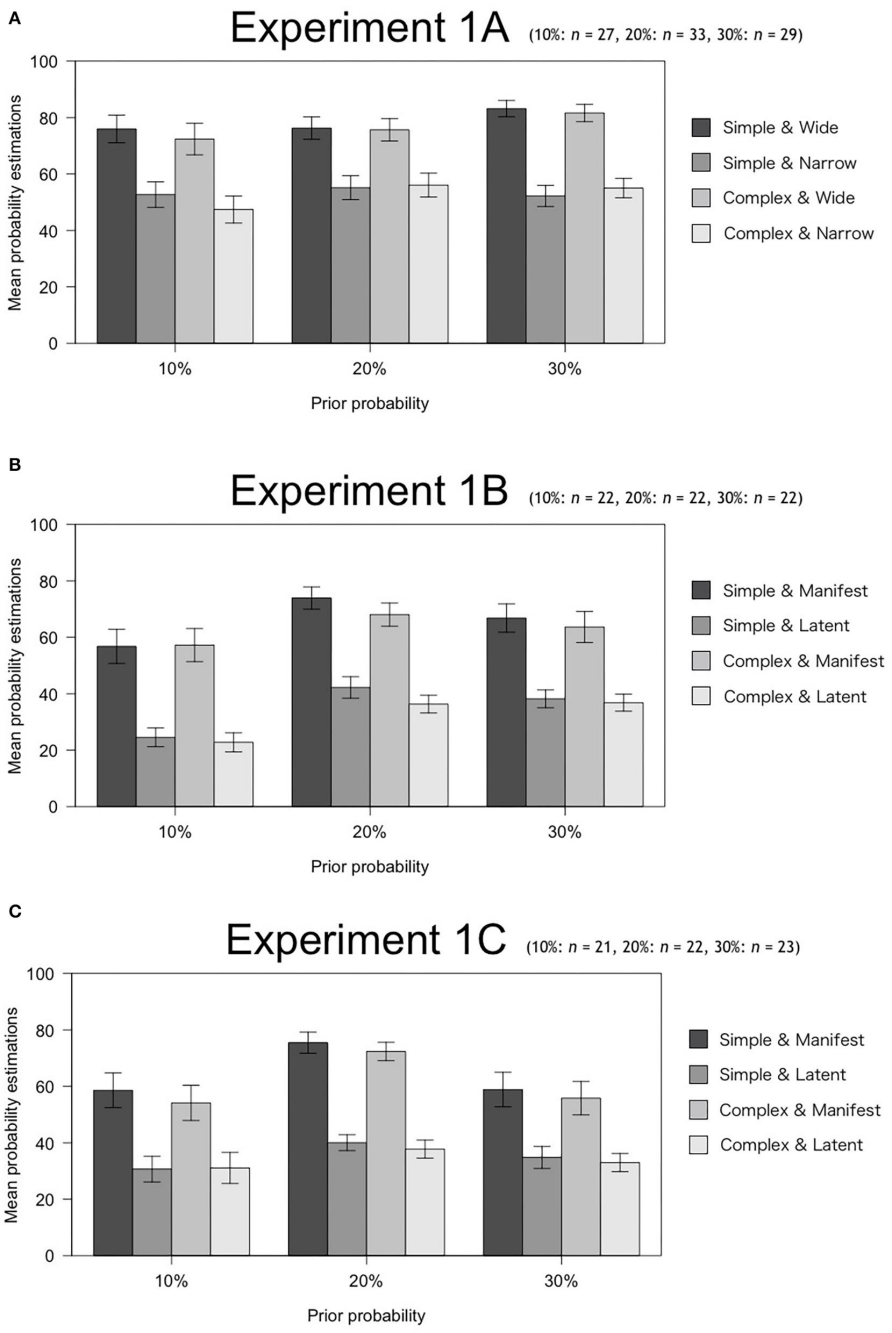
Mean probability estimations with *SE* in Experiment 1.

Figure 1 shows the descriptive statistical values for each experiment. We conducted a three-factor mixed design ANOVA with 2 (Simplicity: Simple, Complex) × 2 (Scope: Wide, Narrow or Latent, Manifest) × 3 (Prior probability: 10, 20, 30%) in order to examine (i) the interaction between Simplicity and Scope; and (ii) the degrees to which Simplicity, Scope, and Prior probability affect estimations of subjective posterior probability.

Table 2 shows the results. First, no interaction between Simplicity and Scope was found in any of the experimental situations (Exp. 1A: *p* = 0.680, Exp. 1B: *p* = 0.977, Exp. 1C: *p* = 0.688). Regarding estimations of subjective posterior probability, Experiments 1A and 1B showed that Scope and Prior probability had significant effects, while Experiment 1C showed that Scope had a significant effect (*p*_*all*_ < 0.001). On the other hand, Simplicity had no significant effect in any of the experimental situations (Exp. 1A: *p* = 0.588, Exp. 1B: *p* = 0.259, Exp. 1C: *p* = 0.408). As mentioned above, Scope (the number of explainable events or the presence or absence of Latent scope) is a causal structure of explanation; hence, it corresponds to explanatory virtue. Therefore, Scope is a factor affecting estimation of subjective posterior probability of causal explanations in all situations among the exploratory virtues considered in this experiment.

**Table 2:** The results of ANOVA in Experiment 1.

	**Factor**	***F-value* (*df*)**	***p***	***η*_*G*_^2^**
Exp. 1A	Simplicity	0.295 (1, 88)	0.588	0.009
	Scope	68.883 (1, 88)	0.001^***^	0.2
	Prior probability	5.241 (2, 87)	0.006^**^	0.03
	Simplicity × Scope	0.171 (1, 88)	0.680	0.001
Exp. 1B	Simplicity	1.281 (1, 65)	0.259	0.005
	Scope	141.781 (1, 65)	0.001^***^	0.554
	Prior probability	12.034 (2, 64)	0.001^***^	0.047
	Simplicity × Scope	0.001 (1, 65)	0.977	0.001
Exp. 1C	Simplicity	0.687 (1, 69)	0.408	0.003
	Scope	95.035 (1, 69)	0.001^***^	0.371
	Prior probability	0.324 (2, 68)	0.570	0.001
	Simplicity × Scope	0.161 (1, 69)	0.688	0.001

** *p < 0.010*,

*** *p < 0.005*.

We also evaluated the effect sizes of the factors, Scope and Prior probability, derived in each experimental situation according to the proposed guideline by Cohen (1988) (small: η_*G*_^2^ = 0.01, medium: η_*G*_^2^ ≥ 0.06, large: η_*G*_^2^ ≥ 0.14). As a result, in all situations, the effect sizes of scope exceeded the large criterion (Exp. 1A: η_*G*_^2^ = 0.200, Exp. 1B: η_*G*_^2^ = 0.554, Exp. 1C: η_*G*_^2^ = 0.371). The effect sizes of prior probability, on the other hand, were all small (Exp. 1A: η_*G*_^2^ = 0.030, Exp. 1B: η_*G*_^2^ = 0.047). Therefore, the effect of scope on the subjective posterior probability estimates was relatively large.

### Discussion

#### Effects of Virtues

In our experiments, joint probability of two causes was explicitly presented. Our results confirmed that Simplicity had no effect while Scope had a large effect on subjective posterior probability. We consider our results to be consistent with those of previous studies [Experiment 1A: Johnson et al. (2014b); Experiment 1B & 1C: Khemlani et al. (2011), Experiment 1B; Johnson et al. (2014b), Johnston et al. (2017)].

Moreover, there was no effect of Simplicity. However, this experiment did not reveal why the effect of Simplicity was absent. Therefore, we examined which of the following is the cause in Experiment 2.

- Participants had no knowledge of Simplicity and therefore did not see the impact of Simplicity.
- Participants had knowledge of Simplicity but did not use it as a criterion for evaluation, so there was no effect of Simplicity.

First, there is the possibility that participants could not use an explanatory virtue such as Simplicity as the evaluation criterion because they did not have the knowledge. We expected that the participants in this case would be affected by the knowledge about the concept of virtue if it was given to him. This is because knowledge and understanding of causal structure and its strength have been shown to affect performance on reasoning tasks, such as categorization of causes or consequences and learning and probability estimation of their relationships (Griffiths et al., 2004; Goodman et al., 2009; Johnson et al., 2014a). In particular, Shultz (1982) argues that knowledge of causal mechanisms imposes two constraints on human causal reasoning, one of which is the impression that certain causal structures are more likely than others (Griffiths et al., 2004). It has also been claimed that human estimations of probability rely heavily on knowledge of causal structures (Cheng, 1997; Griffiths and Tenenbaum, 2005; Lu et al., 2008; Bes et al., 2012). These points will be discussed below with regard to Experiment 2. Also, there is the possibility that participants had knowledge of the concept of Simplicity, but they did not use it as an evaluation criterion. We expected that the participants in this case would not be affected by the knowledge about the concept of virtue even if it was given to them.

It is worth noting that we could not discuss the interaction between Simplicity and Scope because we could not confirm the main effect of Simplicity in Experiment 1. We will also investigate this point in Experiment 2.

#### Effect of Prior Probability

In Experiments 1A and 1B, Prior probability had an effect on the estimation of subjective posterior probability. Specifically, the higher the Prior probability was, the higher the subjective posterior probability was. A similar trend was also observed in Experiment 1C, although this trend did not reach statistical significance.

Humans tend to neglect or ignore Prior probability when updating their beliefs in light of new evidence (Tversky and Kahneman, 1973, 1982). Yet the effect of Prior probability could have been strengthened in the current experiment by the fact that we explicitly provided the Prior probability and did not present any other information. As a result, anchoring and adjustment heuristics may have affected participants' estimates of subjective posterior probability [anchoring and adjustment heuristic; (Kahneman, 2003)]. This refers to a strategy for estimating posterior probability by adjusting it based on previously stored information. In the present experiment, we consider that subjective posterior probability was estimated by adjusting the Prior probability. This refers to a strategy of updating and adjusting the initial information by considering the newly obtained information. However, based on the initial impression of the information, we tend to adjust the initial information. As a result, the value would still be relatively small after the update if the initial value is small, and if the initial value is large, the value would still be relatively large after the update. Therefore, we believe that participants estimate the subjective posterior probability on the basis of the prior probability presented initially in this study. In fact, participants may have estimated the subjective posterior probability on the basis of the initial presented prior probability because the magnitude of the prior probability was proportional to the magnitude of the subjective posterior probability.

## Experiment 2

Experiment 2 aimed to investigate two matters: (i) whether an effect of Simplicity on the estimation of subjective posterior probability emerges if participants have prior knowledge of the explanatory virtues; and (ii) whether there is an interaction between Simplicity and Scope. The definitions of explanatory virtues used in this experiment were the same as those used in the previous experiment. This experiment also consisted of three situations, Exp. 2A, 2B, and 2C, to the same of Experiment 1.

With respect to (i), we set up the following hypotheses. The first hypothesis was that the participants in Experiment 1 did not have knowledge of the concept of Simplicity; therefore, Simplicity did not affect estimating the subjective posterior probability. In this case, we expected that Simplicity affected the estimation of the subjective posterior probability of an explanation when the concept of Simplicity was instructed to participants, as in previous studies. The second hypothesis was that the participants had knowledge of the concept of Simplicity but did not use Simplicity to estimate the subjective posterior probability. We expected that instruction of the concept of Simplicity did not affect the estimation of the subjective posterior probability of the explanation in this case because participants did not use Simplicity as an evaluation criterion, even if they had knowledge about it as in Experiment 1.

### Method

#### Participants

In this experiment, 211 undergraduate students at Nagoya University participated. They did not major in psychology or medical science and were recruited during lectures. Hence, 88 students in Experiment 2A (43 men and 45 women, *M*_*age*_ = 18.72 years, *SD* = 0.88), 61 students in Experiment 2B (45 men and 16 women, *M*_*age*_ = 20.05 years, *SD* = 0.92), and 62 students in Experiment 2C (50 men and 12 women, *M*_*age*_ = 19.77 years, *SD* = 0.71) participated in exchange for course credit. Participants were informed that even if they were in the middle of the experiment, they could cancel their participation if they experience anxiety during this experiment.

The sample size was determined using the same method as in the previous experiment such that, in all experimental situations, the test power was >0.8 (Exp. 2A: *Power(1–*β *error probability)* = 0.960, Exp. 2B: *Power(1–*β *error probability)* = 0.867, Exp. 2C: *Power(1–*β *error probability)* = 0.873).

#### Design, Materials, and Procedure

We used the same experimental design and task as in the previous experiment. Moreover, we used the same explanations in Experiment 1A for Experiment 2A, Experiment 1B for Experiment 2B, and Experiment 1C for Experiment 2C (see Table 1). This time, however, in each scenario, the causal structure of each explanation was provided so that the participants would understand the structure of the explanations more explicitly.

A summary of the concept of explanatory virtues was provided along with the description of each experiment and participants were asked to follow the same procedure as in the previous experiment. Specifically, participants in Experiment 2A were taught about Simplicity and Scope, while participants in Experiments 2B and 2C were taught about the concepts of Simplicity and Latency. Participants read the four explanations, which are the same content corresponding to experimental situations, after being taught those concepts. As mentioned above, for instance, participants in Experiment 2A read the same explanations as Experiment 1A. Then, they estimated, compared, and answered the subjective posterior probabilities of four explanations. It should be noted that the instructional text contained only objective information about the concepts and did not identify what kind of causal structure makes an explanation better (see Table 3).

**Table 3:** Contents of instruction texts used in Experiment 2.

	**Simplicity (Exp. 2A, 2B, & 2C)**		**Scope (Exp. 2A)**		**Latent (Exp. 2B & 2C)**	
	(1)	(2)	(1)	(2)	(1)	(2)
Structures	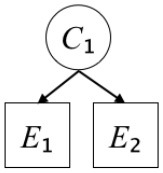	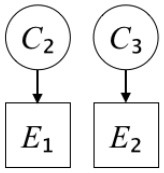	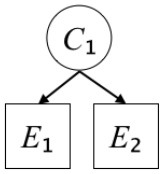	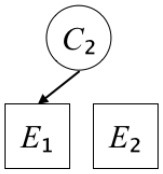	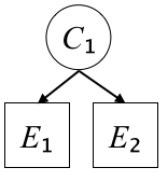	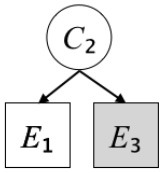
Text	One of the proper**t**ies of an explanation is the concept of “Simplicity.” Simplicity is defined as follows: the smaller the number of causes used to explain a given number of events, the higher the Simplicity. Consider the following situation. C refers to the cause, and E to the event. Explanation 1 explains two events (E1, E_2_) by a single cause (C1). Explanation 2 explains two events (E1, E_2_) by two causes (C_2_, C3). In this case, Explanation 1 has a higher Simplicity.		One of the properties of an explanation is the concept of “Scope.” Scope is defined as follows: the more events that a given number of cause(s) explains, the wider the explanation's Scope. Consider the following situation. C refers to the cause and E to the event. Suppose that two events, E1 and E_2_, are actually observed. In Explanation 1, C1 explains both events (E1 and E_2_). In Explanation 2, C_2_ explains one event (E1), but does not explain E_2_. In this case, Explanation 1 has a wider Scope.		One of the properties of an explanation is the concept of “Scope.” Scope falls on a continuum between “Manifest” and “Latent.” Consider the following situation. C refers to the cause and E to the event. Cause C1 invokes E1 and E_2_. Cause C_2_ invokes E1 and E3. Suppose that two events (E1, E_2_) were actually observed while E3 was not observed. Explanation 1 therefore explains all events that were observed; the Scope of Explanation 1 is Manifest. Explanation 2 explains an event that were observed (E1), but involves an unobserved event (E3); the Scope of Explanation 2 is Latent.	

### Results

**Figure 2:**
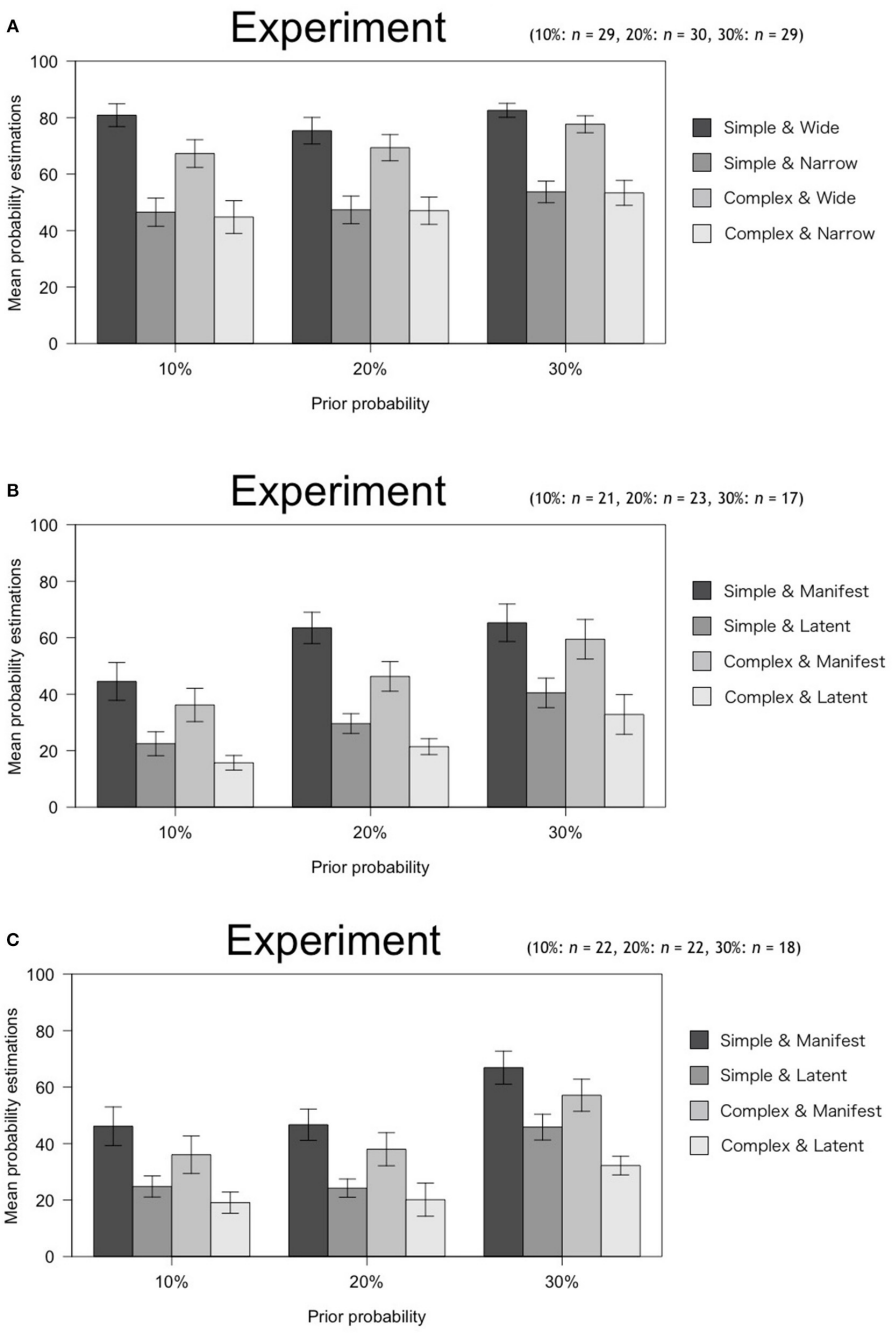
Mean probability estimations with *SE* in Experiment 2.

Figure 2 shows the descriptive statistical values for these experimental situations. As in Experiment 1a, we conducted a three-factor mixed design ANOVA with 2 (Simplicity: Simple, Complex) × 2 (Scope: Wide, Narrow; Latent, Manifest) × 3 (Prior probability: 10, 20, 30%).

Table 4 shows the results. First, Experiment 2 showed that Simplicity, Scope, and Prior probability affected estimations of subjective posterior probability. This means that the effect of Simplicity on estimations of subjective posterior probability emerges only when participants know about the concept of Simplicity as an explanatory virtue and causal structure before making their assessment. Even so, the effect size of Simplicity was smaller than that of Scope in all experiments. Specifically, according to the criteria set by Cohen (1988), the effect size of Scope exceeded the medium or large criterion in all experiments (Exp. 2A: η_*G*_^2^ = 0.302, Exp. 2B: η_*G*_^2^ = 0.070, Exp. 2C: η_*G*_^2^ = 0.198). On the other hand, the effect size of Simplicity was either less than or equal to the small criterion (Exp. 2A: η_*G*_^2^ = 0.008, Exp. 2B: η_*G*_^2^ = 0.017, Exp. 2C: η_*G*_^2^ = 0.033). Furthermore, although Simplicity has an effect, this effect had no interaction with Scope.

**Table 4:** The results of ANOVA in Experiment 2.

	**Factor**	***F value* (*df*)**	***p***	***η*_*G*_^2^**
Exp. 2A	Simplicity	2.907 (1, 87)	0.089^†^	0.008
	Scope	103.982 (1, 87)	0.001^***^	0.302
	Prior probability	4.649 (2, 86)	0.032^*^	0.014
	Simplicity × Scope	1.976 (1, 87)	0.161	0.006
Exp. 2B	Simplicity	4.203 (1, 60)	0.042^*^	0.017
	Scope	16.561 (1, 60)	0.001^***^	0.07
	Prior probability	9.963 (2, 59)	0.002^***^	0.042
	Simplicity × Scope	1.246 (1, 60)	0.265	0.005
Exp. 2C	Simplicity	8.015 (1, 61)	0.005^**^	0.033
	Scope	47.466 (1, 61)	0.001^***^	0.198
	Prior probability	24.159 (2, 60)	0.001^***^	0.101
	Simplicity × Scope	0.118 (1, 61)	0.731	0.002

† *p < 0.100*,

* *p < 0.050*,

** *p < 0.010*,

*** *p < 0.005*.

As the table above shows, this experiment revealed two main findings. First, providing participants with instruction on the concept of explanatory virtues increases the effect of these virtues on estimations of subjective posterior probability, especially the effect of Simplicity, which was not detected in Experiment 1. However, the effect of Simplicity was smaller than that of Scope. Second, as in the previous experiment, there was no interaction between Simplicity and Scope.

### Discussion

#### Effects of Virtues and Their Independence

Experiment 2 did not show any interaction between Simplicity and Scope. This suggests that these two explanatory virtues have independent effects on estimations of subjective probability.

Moreover, in contrast to Experiment 1, the current experiment showed the main effect of Simplicity in all conditions. However, according to Cohen's criterion as mentioned above, Simplicity in all conditions had a small effect, which means that the effect of Simplicity on estimations of subjective posterior probability is smaller than that of Scope.

#### Effects of Instruction

Based on prior research, we initially predicted that the Simplicity factor would affect subjective posterior probability. Surprisingly, however, Experiment 1 failed to confirm this prediction. To determine if the reason is the participants' lack of knowledge about Simplicity or their knowledge but not using the Simplicity criterion, we conducted Experiment 2. Therefore, in Experiment 2, we explicitly instructed participants about the concept of Simplicity prior to the experiment. As a result, an effect of Simplicity on the estimation of subjective posterior probability was found in all situations considered in Experiment 2. This indicates that participants used their newfound understanding of explanatory virtues as a guideline for their evaluation of causal structure and their estimations of subjective posterior probability.

The important point here is that, although we introduced the concept of Simplicity, we did not mention the nature of its effects, specifically, the fact that a simple explanation is considered a better explanation. This indicates that participants will be able to make normative estimates of posterior probability if they are alerted to the importance of Simplicity. Therefore, it is possible that the participants in Experiment 1 did not have knowledge of the concept of Simplicity and could not use it as an evaluation criterion. The knowledge of the concept of Simplicity in Experiment 2 would have no effect if the participants in Experiment 1 did not use it as a criterion for estimating the subjective posterior probability although having knowledge of the concept of Simplicity.

In the context of causal explanations, it has been suggested that prior knowledge about such topics as causal structure affect the estimation of posterior probability (Griffiths and Tenenbaum, 2009). In this study, participants who were given prior knowledge of causation through instruction from the experimenters were aware of the importance of explanatory structure as an evaluation criterion and used this information to assess posterior probability. This result implies that the effect of Simplicity on the estimation of subjective posterior probability may emerge because of conceptual knowledge that is given prior to the assessment.

## General Discussion

### Findings and Novelties of This Study

In this study, we examined how Simplicity and Scope affect estimations of subjective posterior probability, and whether the degree of their effects changes depending on knowledge of the abovementioned explanatory virtues.

To date, it has not been possible to examine the interaction between Simplicity and Scope because no previous studies have examined multiple explanatory virtues in a single experiment. Even in studies that examined a single explanatory virtue, such as Latent scope, it was difficult to measure the effect of Latent scope on probability estimation because the number of effect(s) predicted by the cause(s) differed among studies and because not all studies have shown comparable effect sizes.

The contributions of this study can be summarized as follows. First, we examined the existence of the interaction between Simplicity and Scope by systematically manipulating the combination of these explanatory virtues. Second, we examined how explicit instruction about the concept of explanatory virtues changes the effects of the virtues on estimations of subjective posterior probability.

As for the first point, no interaction between Simplicity and Scope was observed, which means that those virtues affect estimations of subjective posterior probability independently. This finding is important because, in previous studies on explanatory virtues, Simplicity and Scope have only been considered independently. This indicates that there has been an implicit assumption that both virtues independently affect the explanatory variables. The current study gives experimental evidence that confirms the validity of the findings of previous studies in this research area. Moreover, the results of our two experiments showed that the effect of Scope was observed for all conditions, while the effect of Simplicity was either smaller than that of Scope or not seen at all. Thus, it can be confirmed that Scope has a stronger effect on the estimation of subjective posterior probability.

As for the second point, the effect of Simplicity did not emerge if participants had no prior knowledge of the causal structure of an explanation. Its effect did emerge, however, if they were provided with such knowledge before making their assessment. Even in such cases, however, the effect of Simplicity was much smaller than that of Scope.

In addition to probability estimation, the effect of Simplicity has been confirmed in a variety of other contexts, including category-based reasoning (Johnson et al., 2015), social categorization (Johnson et al., 2016b), state-of-mind reasoning (Johnson et al., 2016a), and decision making (Johnson et al., 2016d). All of the above examples show that humans prefer simpler explanations and perform better when given them. This tendency in human thought is called simplicity bias and is thought to be favored because it reduces the cognitive load required for humans to calculate things or make complex decisions, such as Bayesian inferences and categorizations (Feldman, 2016). Lagnado (1994) has also shown that humans prefer simpler explanations. This demonstrates that Simplicity influences evaluations of explanatory goodness.

Based on the above insight, in this study, we hypothesized that simple explanations would positively affect estimations of subjective posterior probability; in Experiment 1, however, this hypothesis was not confirmed. The reason for the absence of a Simplicity effect is interpreted as follows. Previous studies have claimed that the effect of Simplicity can only be seen in situations where prior probability or likelihood is indeterminate [effect in situations where prior probability is indeterminate: Lombrozo (2007); effect in situations where likelihood is indeterminate: Johnson et al. (2014a), Johnson et al. (2019)]. In Experiment 1, we explicitly conveyed the Prior probability to each participant. Furthermore, although there was no mention of likelihood in the scenario, it is possible that the participants may have considered the explanations' likelihood to be 100% because the disease could have been interpreted as definitely responsible for the symptoms. As a result, the effect of Simplicity on Prior probability and likelihood would have been suppressed.

### Contributions to Social Problems

Artificial intelligence (henceforth “AI”) has recently been used for finding out why an event occurred. For example, AI can diagnose with high accuracy diseases a patient has. Even if AI suggests a certain disease as the cause of a certain symptom, it is difficult for humans to understand why AI gave such a diagnosis because AI's algorithm is complexity. The difficulty in understanding why AI suggests a certain disease as the cause of a certain symptom is a problem for using AI.

Machine learning models that can explain the basis for the estimation (Explainable AI; henceforth, “XAI”) have been developed in order to solve this problem. Some studies pointed out the need for using knowledge on social science, especially in psychology, to develop XAI (Confalonieri et al., 2019; Miller, 2019). However, when examining what is a good explanation, almost none of the research and practices on XAI have been grounded on psychological findings (Miller et al., 2017). The findings of this study may provide useful foundational information for this issues.

### Limitations and Future Directions

#### Domain Effect

This study limited the domain of explanation to medical diagnosis. However, individual humans can hold distinct beliefs about what kind of explanation is most plausible in each domain (Shipley, 1993; Kemp et al., 2010). For instance, it has been suggested that humans believe that physical events have simpler causations than social events (Strickland et al., 2016) and that social explanations may require complex causal inferences (Johnson et al., 2019). Moreover, explanations in the domain of biology, especially evolution, are evaluated according to different criteria than those in other domains (Liquin and Lombrozo, 2018).

However, these preceding studies have only considered the effects of a single focused explanatory virtue. Thus, they could not compare the effects of different explanatory virtues nor assess any interactions between those virtues. In the future, it will be useful to examine the effects of explanatory virtues in different domains and to discuss the commonality and individuality of explanatory virtues. For this purpose, the research paradigm devised for the current study may be useful.

#### Trade-off Between Simplicity and Scope

In the real world, situations may arise in which humans do not necessarily regard the simplest explanation as the most likely. This is because we can explain more events by assuming many causes. In other words, there is a trade-off between Simplicity and Scope whereby Scope becomes narrower as the Simplicity of an explanation increases. Therefore, in future work, it may be interesting to search for the equilibrium point of this trade-off between different virtues.

## Conclusion

This study examines how two exploratory virtues, Simplicity and Scope, affect estimations of subjective posterior probability, and how knowledge of these concepts affects the degree of their effects.

Our results show that these explanatory virtues independently affect probability estimations. These results confirm the validity of previous studies suggesting that each explanatory virtue has its own independent effect without examining the interaction between the explanatory virtues. It is also shown that Scope is the main factor explaining estimates of the subjective posterior probability of causal explanations. This result partially supports Lipton's claim that Loveliness can be a guide to Likeliness. Contrary to our expectations, however, Simplicity showed little to no effect. We propose that the reason for this is that the effect of Simplicity was suppressed by our explicit statement of joint probability. However, the effect of Simplicity increased when participants were given knowledge of explanatory structure. This result is consistent with the claims that prior knowledge of causal structure affects human preferences for particular explanations as well as probability estimations.

## Data Availability Statement

The raw data supporting the conclusions of this article will be made available by the authors, without undue reservation.

## Ethics Statement

The studies involving human participants were reviewed and approved by the Ethics Committee of Graduate school of Informatics, Nagoya University. Written informed consent from the participants' legal guardian/next of kin was not required to participate in this study in accordance with the national legislation and the institutional requirements.

## Author Contributions

AS mainly contributed above-mentioned works. KM and HT corrected the manuscript. All authors designed the study, conducted the experiment and performed data collection, data analysis and data interpretation, and wrote the manuscript.

## Conflict of Interest

The authors declare that the research was conducted in the absence of any commercial or financial relationships that could be construed as a potential conflict of interest.
